# In vivo assessment of mitochondrial respiratory alternative oxidase activity and cyclic electron flow around photosystem I on small coral fragments

**DOI:** 10.1038/s41598-020-74557-0

**Published:** 2020-10-15

**Authors:** Félix Vega de Luna, Juan José Córdoba-Granados, Kieu-Van Dang, Stéphane Roberty, Pierre Cardol

**Affiliations:** Inbios/PhytoSystems, Université de Liège, 4000 Liège, Belgium

**Keywords:** Bioenergetics, Photosynthesis, Plant symbiosis, Respiration, Biophysical methods, Coral reefs

## Abstract

The mutualistic relationship existing between scleractinian corals and their photosynthetic endosymbionts involves a complex integration of the metabolic pathways within the holobiont. Respiration and photosynthesis are the most important of these processes and although they have been extensively studied, our understanding of their interactions and regulatory mechanisms is still limited. In this work we performed chlorophyll-*a* fluorescence, oxygen exchange and time-resolved absorption spectroscopy measurements on small and thin fragments (0.3 cm^2^) of the coral *Stylophora pistillata*. We showed that the capacity of mitochondrial alternative oxidase accounted for ca. 25% of total coral respiration, and that the high-light dependent oxygen uptake, commonly present in isolated Symbiodiniaceae, was negligible. The ratio between photosystem I (PSI) and photosystem II (PSII) active centers as well as their respective electron transport rates, indicated that PSI cyclic electron flow occurred in high light in *S. pistillata* and in some branching and lamellar coral species freshly collected in the field. Altogether, these results show the potential of applying advanced biophysical and spectroscopic methods on small coral fragments to understand the complex mechanisms of coral photosynthesis and respiration and their responses to environmental changes.

## Introduction

Coral reefs owe most of their high diversity and productivity to energetic relationships occurring in Scleractinian corals^1^. Coral symbiotic nature is characterized by a complex metabolism integration between the cnidarian host and its photosynthetic endosymbiont (Symbiodiniaceae, dinoflagellate)^2,3^. The coral physiology is thus determined by multiple metabolic traits^4^ from which respiration and photosynthesis are the utmost driving processes^5^. Coral bioenergetics has been investigated through gas exchange analysis thanks to the development of different systems which allow measurements in situ (e.g.^6,7^) or in laboratory (e.g.^8,9^). Coral respiration is largely fuelled by photosynthetically-derived carbon molecules^10^, by active heterotrophy^11^, and by oxygen released during photosynthesis^12^. It is carried out in both animal and algal mitochondria by the respiratory complexes (Complex I, II, III, IV and F_1_F_O_ ATP synthase) which fulfils most of the cellular ATP demands. Host mitochondrial electron transport activity is directly related to respiratory substrates availability^13^. However, it has been suggested that the way in which corals consume storage molecules, as lipids, may be related to their sensitivity to bleaching conditions^14^. In the model sea anemone *Exaiptasia pallida*, thermal stress causes a decrease in gene expression of cytochrome *c* and ATP synthase subunit *a*, and ultimately to mitochondria degradation and apoptosis^15^. Cnidarian cellular physiology seems to be intimately linked to mitochondrial redox state^16^. Beyond this, the role of non-proton pumping (and consequently non-ATP yielding) alternative respiratory enzymes, such as alternative NADH dehydrogenases and alternative ubiquinol oxidase (AOX) which genes are present in the cnidarian genome^17,18^, is still unknown. Differently from the host, Symbiodiniaceae mitochondria lack the canonical NADH:ubiquinone oxidoreductase (Complex I) but possess an AOX^19,20^. Understanding the contribution of all these components to symbiont and host respiration will help to characterize the physiological responses of corals to natural oxygen variations in the reefs and its interaction with stress^21^.

Coral photosynthesis is carried out by Symbiodiniaceae algae only. Light absorption occurs in the thylakoid antenna system, which in peridinin plastid-containing algae as Symbiodiniaceae consists of a membrane embedded chlorophyll *a*-chlorophyll *c*_2_-peridinin protein complex (acpPC) and a water soluble peridinin-chlorophyll *a*-protein complex (PCP)^22^. These antennas transfer energy to photosystem II (PSII) and photosystem I (PSI) reaction centers, allowing charge separation, and a linear electron flow (LEF) from oxidation of water molecules at the PSII donor side to reduction of NADP^+^ at the PSI acceptor side. The intermediate Cytochrome *b*_*6*_*f* complex oxidises the plastoquinol pool (electron acceptor of PSII) and reduces cytochrome *c*_*6*_ (cyt *c*_*6*_; electron donor of PSI), meanwhile translocating protons to the lumenal side of the thylakoid. This transmembrane electrochemical proton gradient is then used by the CF_1_F_O_ ATP synthase complex. The availability of NADPH and ATP for CO_2_ fixation depends largely on a complex balance between the different processes producing and consuming the electrochemical proton gradient, ATP and NADPH. An alternative cyclic electron flow (CEF), involving PSI and Cytochrome *b*_*6*_*f*, may work to balance the energetic requirements for CO_2_ fixation. CEF activity, by contributing to increase the electrochemical proton gradient, accomplishes two main functions that are driving the synthesis of extra ATP and inducing a non-photochemical quenching (NPQ) dissipation mechanism that may prevent PSII photoinhibition^23^. CEF has been poorly studied in Symbiodiniaceae and its role under steady physiological conditions, both in culture and *in hospite*, is not clear.

Carbon fixation is carried out by a type II Rubisco which is characterized by a low CO_2_-O_2_ selectivity factor, and requires the presence of an efficient CO_2_ concentrating mechanism^24,25^ largely controlled by the host^26,27^ that limits the occurrence of the photorespiratory pathway^24^. After accomplishing carbohydrate synthesis, an important amount of photosynthetic products is transported to the host^28^ thanks to the expression of membrane transporters^29^. The photosynthetic activity of corals is dynamic at different levels of time and space scales in coral reefs^30^, and it has been shown to be highly coupled with nutrients^31^ and water flow^7^. The delicate balance of symbiosis in corals can be disrupted as a consequence of affections in the photosynthetic activity. For instance, a decrease in the electron transfer at the PSII acceptor side in heat sensitive species, leads to damage in thylakoid membranes and potentially to bleaching^32^. Altogether, this points out the importance of studying Symbiodiniaceae photosynthesis *in hospite*.

At the cellular level, different regulatory mechanisms of photosynthetic electron flow help to balance the light energy absorbed and the energetic requirements for carbon fixation in microalgae^33^. The characterization of these mechanisms has been achieved through in vivo PSII-related chlorophyll *a* fluorescence^34^. This technique has shown that different Symbiodiniaceae isolates show diverse photobiological responses to light changes^35^. For instance, the presence of CEF has been suggested to occur in *Symbiodinium* genus (formerly Clade A) both free-living and in symbiosis^36^. The combination of chlorophyll *a* fluorescence and oxygen exchange measurements also showed that *Symbiodinium* sp. in culture activates a light-enhanced dark respiration during photosynthesis^8^. A more detailed characterization through the combination of various techniques (O_2_ exchange, PSII chlorophyll *a* fluorescence emission, PSI primary electron donor [P700] absorbance change in the far-red spectrum, and analysis of the thylakoid transmembrane electric field), revealed that an oxygen-dependent reduction at the acceptor side of PSI occurs as photoprotective mechanism in *Symbiodinium* isolates^37,38^.

Spectrophotometric measurements of PSI activity performed in the anemone model species *Exaiptasia pallida* harboring *Breviolum* sp. (formerly Clade B) revealed that exposure to high temperature can stimulate the CEF capacity^23^. However, on corals such studies are still scarce because: (1) their morphology is not suitable for most spectroscopic apparatus that accommodate 1 cm^2^ cuvette; (2) the thickness and nature of their calcareous skeleton makes coral branches or nubbins not homogenous spectroscopic objects, scattering and poorly transmitting detecting light; (3) actinic light cannot be delivered in a homogenous manner on all sides of the coral specimens even in simple systems, like in oximeter chambers.

The simultaneous monitoring of in vivo PSI and PSII activity in corals has been attempted by using the pulse-amplitude-modulation technique (Dual-PAM, Walz, Germany) which records variable chlorophyll *a* fluorescence and P700 photo-oxidation. Hoogenboom et al.^39^ analysed the laminar coral *Turbinaria reniformis* and concluded that PSI was not a major site for damage during thermal stress. They also suggested the existence of CEF at high light intensities because of a decoupling between PSII and PSI activity was observed, but no clear results were obtained from the branching coral *S. pistillata*. In contrast, a recent study using improved settings of the Dual-PAM in reflectance mode, in combination with chlorophyll *a* fluorescence kinetics, showed a strong sensitivity of PSI to a Calvin cycle inhibitor and thermal stress in the plate-like coral *Pavona decussata*^40^. This highlights the lack of knowledge on PSI activity in corals in response to environmental changes.

Coral skeleton is composed by aragonite depositions which has a low light absorption capacity and diffuses and scatters light through its structure^41^. Other works have used small coral fragments to better characterize biophysical properties of the coral skeleton^41^ or coral pigments through spectroscopic kinetics^42^. Consequently, coral fragments might be good spectroscopic objects but, despite this, they have not been studied by advanced biophysical and spectroscopic methods commonly applied for other photosynthetic organisms to study photosynthesis (and more generally bioenergetics). In this work we challenged thin and small coral fragments to classical physiological and highly specialized spectroscopic measurements of respiration and photosynthesis, and we aim to propose them as reliable sources of in vivo information.

## Methods

### Biological material

Coral colonies of *Stylophora pistillata* variety Milka were obtained from DeJong Marinelife (Netherlands), and maintained in a 300 L capacity aquarium filled with artificial seawater prepared at 34 PSU (Coral Pro Salt, Red Sea Fish LTD). Temperature was set to 26 °C and light was provided by a LED spot light (A360W tuna Blue, Kessil, USA) at 100 μmol photons m^−2^ s^−1^ in a light–dark cycle of 12 h:12 h. Corals were fed two to three times per week with freshly hatched artemia. Some measurements reported here were also conducted on coral colonies of *Pachyseris speciosa*, *Pocillopora damicornis*, *Acropora formosa* and *Pavona cactus*. These colonies were collected by SCUBA diving in the area of Malakal in the Palau Archipelago (7°19′28.3″ N 134°28′01.6″ E), during a sampling campaign conducted in January 2018. After collection, colonies were transferred to the facilities of the Palau International Coral Reef Center (PICRC) and suspended from a nylon wire inside a tank supplied with running filtered natural seawater. Temperature in the tank was 30 ± 1 °C and the maximum Photosynthetic Photon Flux Density (PPFD) at noon was about 100 μmol photons m^−2^ s^−1^.

*Symbiodinium microadriaticum* culture (CCMP 2467) originally isolated from shallow water coral *S. pistillata* from the Red Sea, was kindly provided by Prof. Oren Levy (Bar Ilan University, Israel). The culture was maintained in 250 mL Fernbach flasks containing 75 mL of artificial sea water (Coral Pro Salt, Red Sea Fish LTD) at 34 PSU and enriched with Guillard’s (F/2) Marine Water Enrichment Solution (Sigma-Aldrich). The culture was grown under cool white fluorescent lamps at 75 µmol photons m^−2^ s^−1^ on a 12 h:12 h light:dark cycle and it was subcultured weekly. For spectroscopic measurements cells were adjusted to 10 µg of total chlorophyll per mL and supplemented with 10% (w/v) of Ficoll to avoid sedimentation.

### Coral fragment preparation

**Figure 1:**
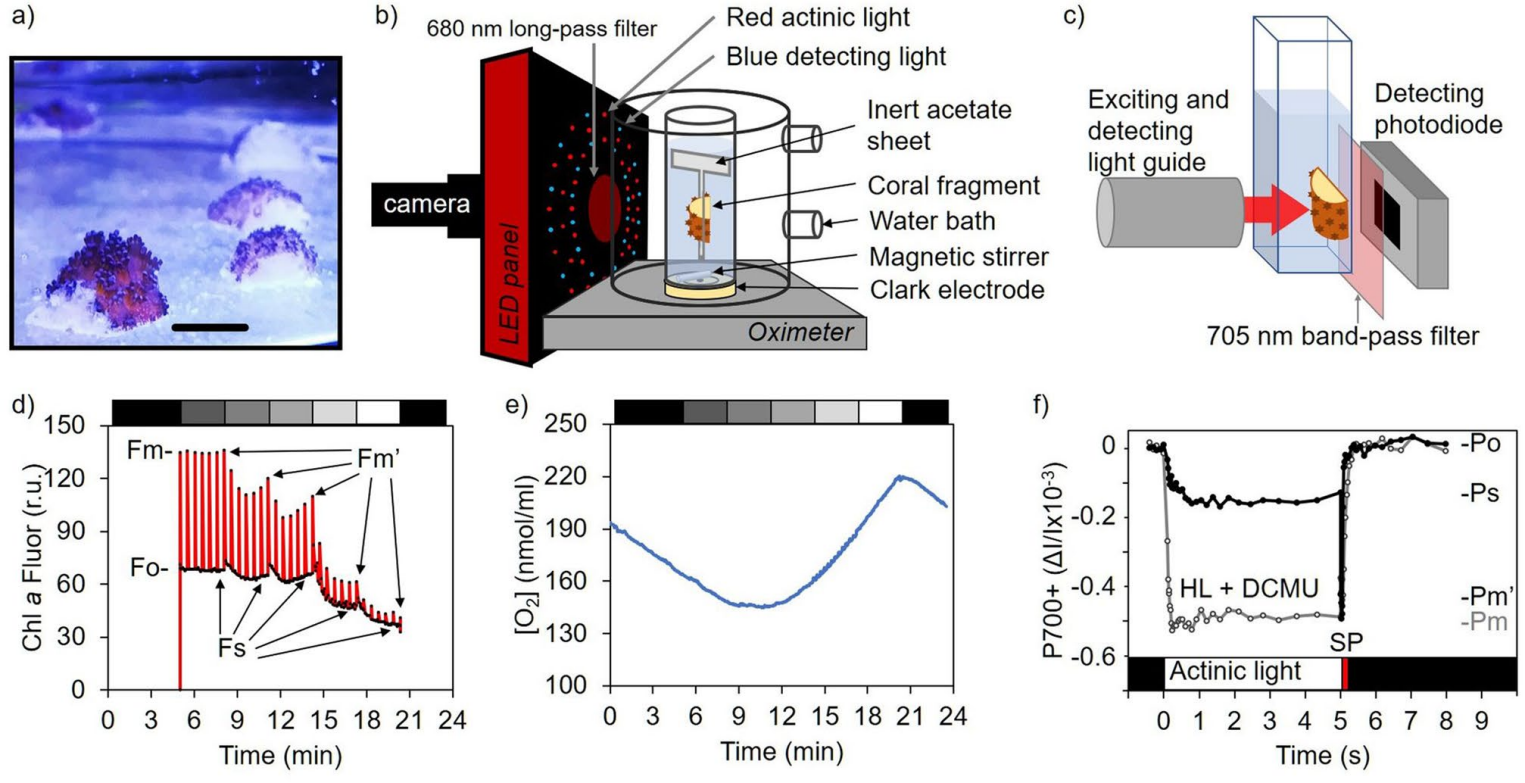
Experimental setup and representative experiments on small and thin coral fragments. (**a**) Picture of recently obtained *S. pistillata* fragments settled back in the aquarium; the black bar indicates 5 mm. (**b**) Schematic representation of the setup used for simultaneous analyses of chlorophyll *a* fluorescence yield and oxygen exchange. (**c**) Disposition and orientation of a coral fragment into a 1 cm^2^ glass cuvette relative to the light guide and the detecting photodiode in the JTS-10 spectrophotometer. Drawing in (**b**) and (**c**) were created using PowerPoint of Microsoft Office 365 ProPlus. (**d**,**e**) Representative records of chlorophyll *a* fluorescence yield measurements and oxygen concentration changes. Each light step (represented by the upper bar with a grey scale) lasted three minutes and saturating pulses were given each 30 s (r.u. = relative units). (**f**) Representative record of Photosystem I primary donor (P700) photo-oxidation upon five seconds of illumination (210 µmol photons m^−2^ s^−1^) followed by a saturating pulse of light (SP), in presence of PSII inhibitor (DCMU at 20 µM).

Square fragments of about 5 by 5 mm (Fig. 1a) were cut from various coral colonies with a Dremel 8220 (Dremel, USA) equipped with a diamond disk. For the branching corals *S. pistillata* and *A. formosa*, two transversal cuts were done close to the end of similar terminal branches, one to remove the growing white tip, and the other which determine the length of the fragment (i.e. approximately 5 mm). A third longitudinal cut determined the thickness of the fragment. The longitudinal cut in the shrubby coral *P. damicornis* was made in the skeleton regions thicker than 3 mm. Laminar corals *P. speciosa* and *P. cactus* were cut perpendicularly to the plane of the skeleton. This procedure was performed out of the aquarium and took less than 30 s per fragment, after which it was rapidly immersed in another sea water container to remove debris. Coral fragments were analysed within one hour after being cut (termed ‘day 0’) or thereafter maintained in the same aquarium conditions as their parent colony.

### Chlorophyll *a* fluorescence measurement

In vivo chlorophyll *a* fluorescence images were obtained by a SpeedZen imaging system (BeamBio/API, France). An array of blue LEDs (450–470 nm) was used to excite chlorophyll *a* during 400 μs, and images were obtained by a CCD camera (UI-3240CP-NIR-GL Rev.2, IDS, Obersulm, Germany) filtered by a long pass red filter (680 nm) (Fig. 1b). After incubating samples in darkness for at least 15 min, minimal chlorophyll *a* fluorescence yield (Fo) image was acquired. A saturating red actinic light pulse (3000 μmol photons m^−2^ s^−1^), provided by a LED array (660 nm), was applied for 200 ms and maximal chlorophyll *a* fluorescence yield (Fm) was acquired. Maximum quantum yield of photosystem II was calculated as Fv/Fm = (Fm − Fo)/Fm^43^.

Coral fragments were exposed to different PPFD (15, 68, 172, 354 and 755 μmol photons m^−2^ s^−1^, provided by the same red LED array than the saturating pulse) during 3 minutes and fluorescence (Fs) was recorded every 5 s (Fig. 1d). A saturating red actinic light pulse was applied to obtain the maximum fluorescence yield under light acclimation (Fm′) every 30 s, and the effective quantum yield of PSII at steady state was calculated as ϕPSII = (Fm′ − Fs)/Fm′. The relative electron transfer rate through PSII (rETR-PSII) was then calculated as ϕPSII x PPFD, and the non-photochemical quenching of chlorophyll a fluorescence (NPQ) as (Fm − Fm′)/Fm′^43^.

Fast induction curves of chlorophyll *a* fluorescence were recorded with a Handy PEA fluorometer (Hansatech Instruments, UK), by illuminating the sample during 10 s with a strong red light pulse (peak wavelength of 650 nm) of 2000 μmol photons m^−2^ s^−1^. This fluorescence rise is characterized by a sudden increase from a basal fluorescence level (O), with two intermediate inflections (J and I), to a maximum fluorescence level (P) commonly referred as OJIP transient (see Chapter 12 in^34^). O phase is taken at 50 μs, J appears at 2 ms, while I appears after 50 ms and P after 2 s in corals. Each coral fragment was set in the bottom of a standard spectrophotometer cuvette facing the head sensor and filled with 2 mL of artificial seawater and maintained in darkness for 15 min before the measurement.

### Light-induced absorption changes

All measurements were performed with a JTS-10 spectrophotometer (BioLogic, France) piloted by a BeamBio/API (France) electronic device. When necessary, the final size of the coral fragments was adjusted to fit into standard 45 mm × 12.5 mm × 12.5 mm (H × W × D) glass spectrophotometer cuvette, i.e. with a pathlength of 10 mm. Each coral fragment was held in the cuvette with the side containing most polyps facing the light source and the skeleton exposed area (when applicable) in front of the detecting photodiode (Fig. 1c). This guaranteed a controlled illumination of the sample.

In the photosynthetic electron transport chain of peridinin chloroplast-harboring dinoflagellates cyt *c*_6_ is the electron donor to PSI instead of Plastocyanin (PC)^44^. For this reason, light-induced absorption changes of P700 were directly measured at 705 nm^45^ by applying μs detecting flashes during short intervals of dark pulses (as described in^46^). Continuous actinic light at different intensities (25, 85, 210, 380, 670 and 1400 μmol photons m^−2^ s^−1^) was provided during 5 s (3 min for field coral specimens) by a LED array (640 nm). When coral fragments were exposed to continuous light, a stable negative change in absorbance at 705 nm (due to the formation of oxidized P700)^47^ was obtained and recorded as Ps (Fig. 1f). A strong saturating pulse of light (9000 μmol photons m^−2^ s^−1^) was applied to obtain a more negative absorbance signal corresponding to maximum photo-oxidation of P700 as Pm’, while after a dark period of 1 s absorbance was recorded as Po. Maximum absorbance change (Pm) was estimated by adding the potent photosystem II inhibitor DCMU (3-(3,4-dichlorophenyl)-1,1-dimethylurea) at 20 μM (Fig. 1f). Photosystem I quantum yield was calculated as Y(I) = (Pm′ − Ps)/(Pm − Po), quantum yield of non-photochemical energy dissipation due to donor side limitation as Y(ND) = (Ps − Po)/(Pm − Po), and the quantum yield of non-photochemical energy dissipation due to acceptor side limitation as Y(NA) = (Pm − Pm′)/(Pm − Po)^48^. The relative electron transfer rate through PSI (rETR-PSI) was calculated as the product of Y(I) and PPFD.

Absorbance changes of some pigments in the green region of the visible spectrum occur in response to light-induced changes of the trans-thylakoid electric field. This so-called electrochromic shift (ECS) is a useful way to determine photosynthetic parameters such as stoichiometry of active PSI and PSII in photosynthetic eukaryotes^49^, including Symbiodiniaceae^37^. ECS spectra were determined by measuring light-absorption changes in response to 2 ms continuous illumination, each 10 nm using light band-pass filters from 480 to 600 nm, as previously described^50^.

The ratio between active PSII and PSI centers can be quantified because the ECS signal responds linearly to the intensity of the electric field^37^. ECS signal at 554 nm was followed by applying a saturating single-turnover flash of 5 ns provided by a Nd:YAG Laser (Minilite II, Continuum) and its amplitude was considered as PSI + PSII reaction centers contribution. PSII inhibitors DCMU (20 µM, from a stock solution dissolved in ethanol) and hydroxylamine (1 mM, from a stock solution dissolved in distilled water) were added simultaneously and incubated in darkness during 10 min, another single-turnover flash was given and the resulting ECS amplitude accounted for PSI contribution. Ten consecutive repetitions, separated by 5 s in darkness, were averaged per measurement.

### Oxygen exchange

Oxygen consumption (respiration) or evolution (photosynthesis) were measured with an Oxygraph + System (Hansatech, UK). Oxygen concentration calibration was carried out at two points with 0.2 µm-filtered F/2 medium either saturated in oxygen after vigorous shaking, or depleted in oxygen by addition of sodium dithionite (Sigma-Aldrich).

Coral fragments were held suspended into a DW1 oximeter chamber (Hansatech, UK) by an inert piece of acetate sheet. This enabled the adequate stirring of the medium by the magnetic stir bar (Fig. 1b). Each fragment was dark acclimated for at least 15 min with the chamber opened before closing it to monitor oxygen consumption in the dark for 5 min. After this dark period, illumination was provided by the Chlorophyll *a* fluorescence imaging system, so during fluorescence recording oxygen concentration was simultaneously acquired (Fig. 1e).

### Chlorophyll content and coral surface determination

Pigments were extracted by incubating coral fragments in cold methanol during at least 12 h in darkness at 4 °C. For *S. microadriaticum*, cells were resuspended in cold methanol and vortexed in presence of 500 µL of acid-washed and autoclaved glass beads (710–1180 µm; Sigma-Aldrich) for 2 min at 30 Hz with a TissueLyser II (QIAGEN). Pigment extracts were centrifuged at 16,000 g for 10 min to remove debris, and chlorophyll *a* and *c*_2_ concentrations were calculated according to^51^ from absorption measurements at 632 and 665 nm. Tissue-containing surface of coral fragments was calculated from conventional real color pictures of each fragment by using the software ImageJ (version 1.51j8).

### Symbiodiniaceae genotyping

Small fragments of each species (n = 3) were removed from independent colonies and immediately processed or preserved in RNA*later* (Invitrogen). Fresh or preserved fragments were washed with filtered artificial sea water and coral tissues were extracted by airbrushing the fragments into sterile PBS. DNA from the microalgal fraction was then extracted by using a DNeasy Plant mini kit (QIAGEN) according to the manufacturer’s instructions, with slight modifications. The PCR amplification of the ITS2 gene marker was performed using primers pair SYM_VAR_5.8S2 and SYM_VAR_REV as detailed in^52^. The amplicons library was constructed using the Nextera XT kit (Illumina, Inc., San Diego, CA, USA) and sequencing was carried out on the Illumina MiSeq platform with 2 × 250 bp read configuration. The ITS2-type profiles were obtained by using the SymPortal analytical framework^53^.

## Results

**Figure 2:**
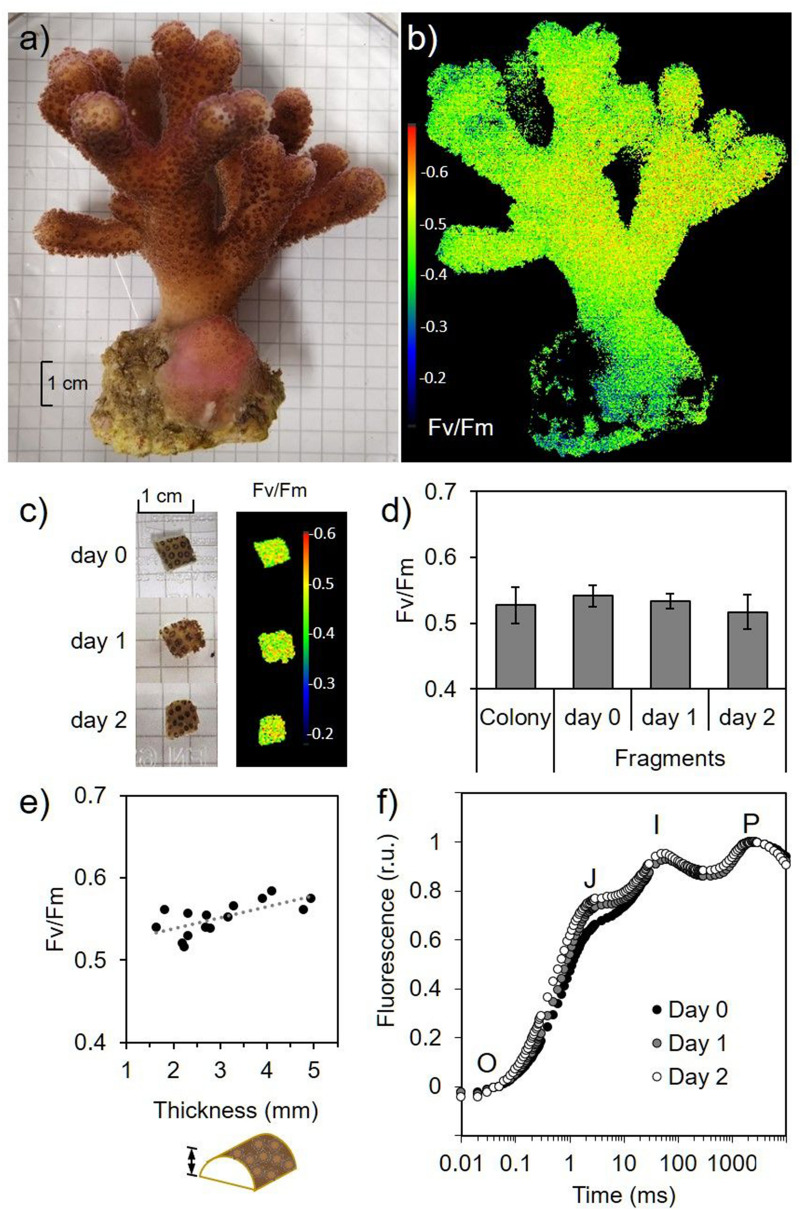
Maximum photosynthetic quantum yield of photosystem II (Fv/Fm) is not altered in thin fragments of the coral *S. pistillata*. (**a**) Picture of one *S. pistillata* colony maintained in aquarium conditions. (**b**) False-color picture of Fv/Fm obtained by chlorophyll *a* fluorescence imaging of Fo and Fm. (**c**) Representative coral thin fragment monitored during two days and its related Fv/Fm false-color image. (**d**) Fv/Fm measured on the apical region of coral colonies where coral fragments were sampled and coral fragments monitored two days after sampling. (**e**) Fv/Fm of coral fragments of variable thickness after one day of sampling. (**f**) Polyphasic chlorophyll *a* fluorescence rise from dark adapted coral fragments was normalized to 0 at 50 µs (‘O’ phase) and to 1 at the plateau (‘P’ phase) (r.u. = relative units). The two typical inflections, phase J and I, are indicated (n = 6 for (**d**) and (**f**)).

Whole *S. pistillata* coral colonies containing *S. microadriaticum* exhibited a homogeneous pigmentation (as illustrated in Fig. 2a). Chlorophyll *a* fluorescence imaging indicated a maximum quantum yield of photosystem II (Fv/Fm) of 0.53 ± 0.03 (Fig. 2b,d). Small half-cylinder shape fragments were cut from these colonies. They were covered with about 35 mm^2^ of coral tissue with an average total chlorophyll content (Chl *a* + *c*_2_) of 30.5 ± 7.6 µg cm^−2^, a Chl *a*/*c*_2_ ratio of 2.39 ± 0.11 and 61 ± 11 polyps cm^−2^ (n = 16). These values remained stable up to 2 days after cutting and no visual sign of tissue necrosis was observed during this period (Fig. 2c). Although there was a positive correlation (R^2^ = 0.41, *p* = 0.007) between Fv/Fm and fragment thickness (from 1.5 to 5 mm), the slope of the linear regression is very small so that the difference between a 2 mm fragment and a 5 mm fragment is less than 6% (Fig. 2e). In addition, the average of Fv/Fm values of coral fragments is not significantly different to the Fv/Fm value of coral colonies (one-way ANOVA, *F*(3,20) = 2.14, *P* = 0.13) (Fig. 2d). The fast polyphasic chlorophyll *a* fluorescence rise (*i.e.* OJIP curves) was obtained from freshly cut coral fragments and from 1- or 2-days old fragments (Fig. 2f) and no changes in the relative amplitude or presence of phases were observed.

**Figure 3:**
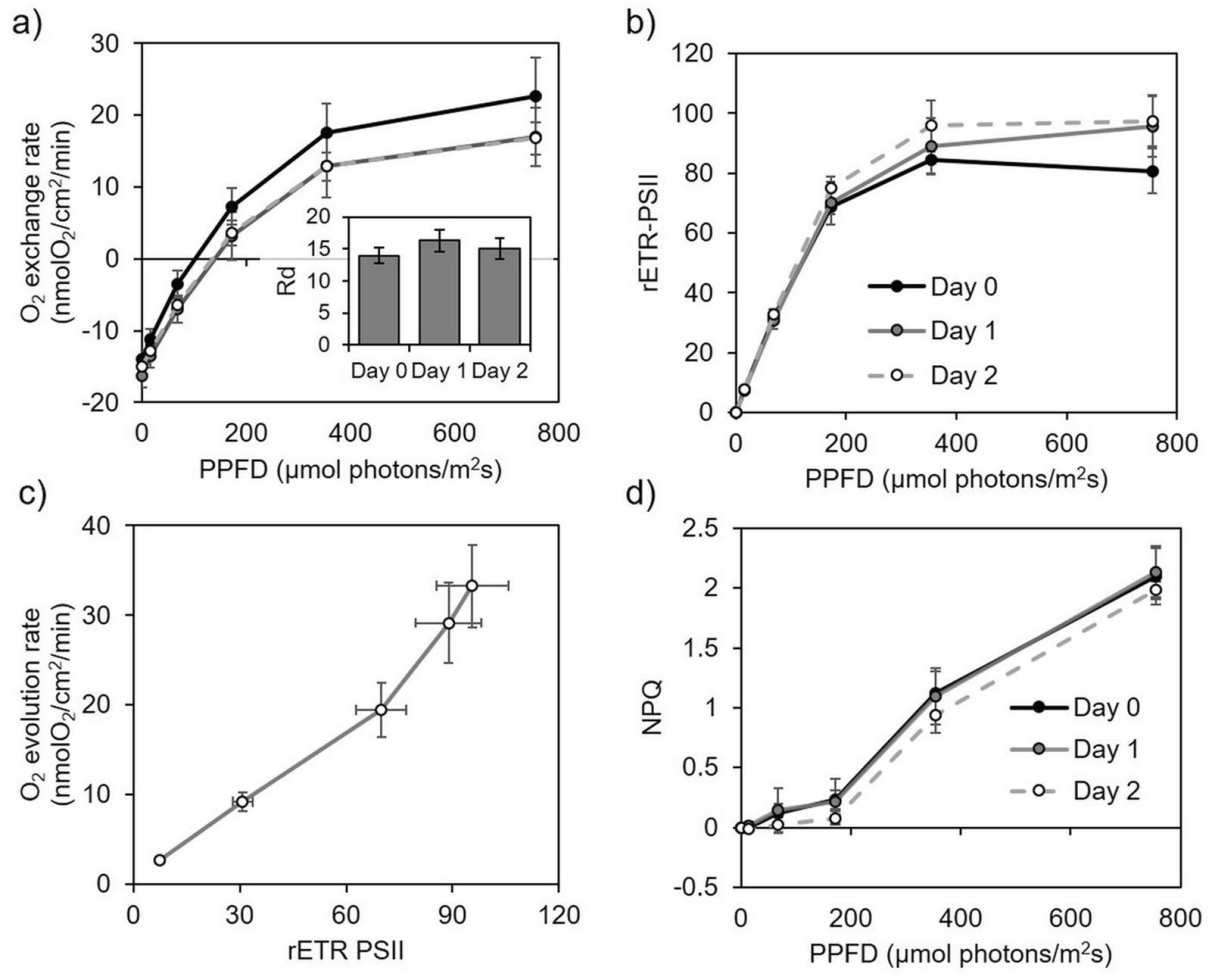
In vivo measurement of respiration and photosynthesis in *S. pistillata* coral fragments. (**a**) inset : Oxygen consumption in the dark (Rd, nmol O_2_ cm^−2^ min^−1^). (**a**,**b**) Light-dependent oxygen exchange and relative electron transport rate (rETR-PSII) were obtained simultaneously. (**c**) Relationship between rETR-PSII and oxygen evolution. (**d**) Non photochemical quenching of fluorescence (NPQ) is shown for three minutes light-acclimated coral fragments. Vertical bars in (**a**–**d**) represent standard deviation, n = 6.

To further test the good health of coral fragments, we determined respiratory and photosynthetic oxygen exchange rates from 3 mm thick fragments. Dark oxygen consumption (Rd) was on average 13.9 ± 2.1 nmol O_2_ cm^−2^ min^−1^ over the three days and remained stable (Fig. 3a, inset). Net oxygen evolution as a function of the light intensity (Fig. 3a) was obtained simultaneously with fluorescence-based calculation of relative electron transport rate of PSII (rETR-PSII) (Fig. 3b). Both parameters showed a similar light dependency as indicated by the fact that rETR-PSII linearly correlated to oxygen evolution rates (R^2^ = 0.98, *p* = 0.002) (Fig. 3c). Moreover, non-photochemical quenching (NPQ) capacity also remained stable (Fig. 3d).

Altogether, these data indicated that the global photosynthetic and respiratory activities of the coral fragments remained stable after cutting. In addition, most of the coral fragments showed the same potential to regenerate the tissue and the coral skeleton, as already shown for fragments of similar size^54^.

**Figure 4:**
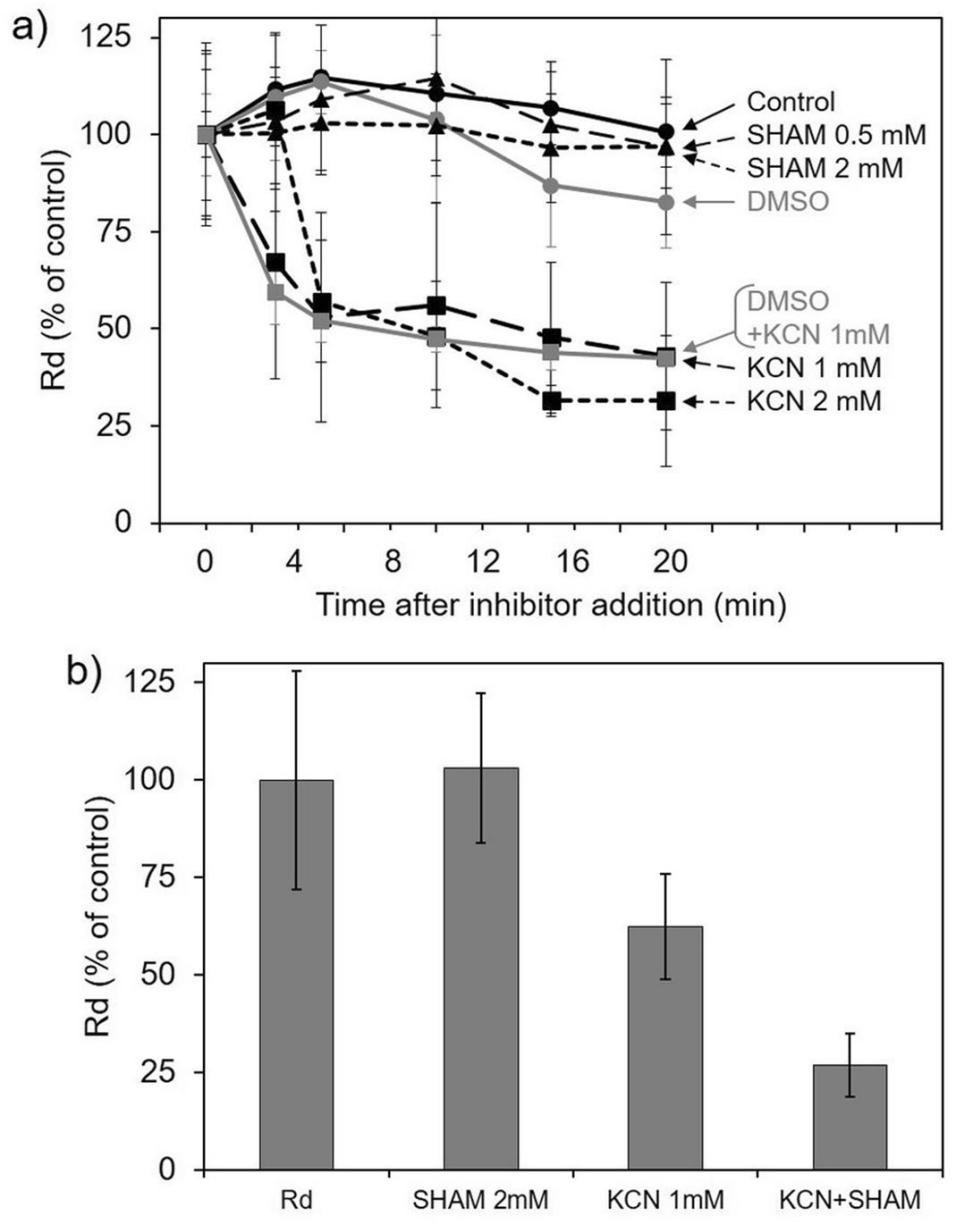
Impact of SHAM and KCN on dark respiration of *S. pistillata* coral fragments. (**a**) Respiratory rates of dark-adapted coral fragments were monitored up to 20 min in dark conditions, n = 3. Cyanide (KCN dissolved in deionized H_2_O) at 1 or 2 mM, n = 4. SHAM (dissolved in DMSO) at 0.5 or 2 mM, n = 4. DMSO at 2%, n = 4. DMSO at 2% plus KCN at 1 mM, n = 6. (**b**) Mitochondrial dark respiration rate (Rd) and the effect of SHAM 2 mM, KCN 1 mM or SHAM 2 mM + KCN 1 mM (KCN + SHAM). Respiratory rates were measured 5 min after the addition of inhibitors, n = 10.

We then tested the effect of two classical inhibitors on respiration: potassium cyanide (KCN), a potent inhibitor of mitochondrial cytochrome *c* oxidase complex (Complex IV), and salicylhydroxamic acid (SHAM), an inhibitor of mitochondrial alternative oxidase (AOX). Addition of 1 or 2 mM KCN reduced Rd to 50% after five minutes (Fig. 4a). Addition of 2 mM SHAM further decreased the remaining cyanide-insensitive oxygen consumption to 26% of total Rd (Fig. 4b), and higher concentrations of SHAM (4 or 6 mM) did not suppress this residual oxygen consumption (data not shown). In contrast, addition of SHAM up to 2 mM alone did not have an impact on Rd during the 20 min of exposure to the inhibitor (Fig. 4a).

**Figure 5:**
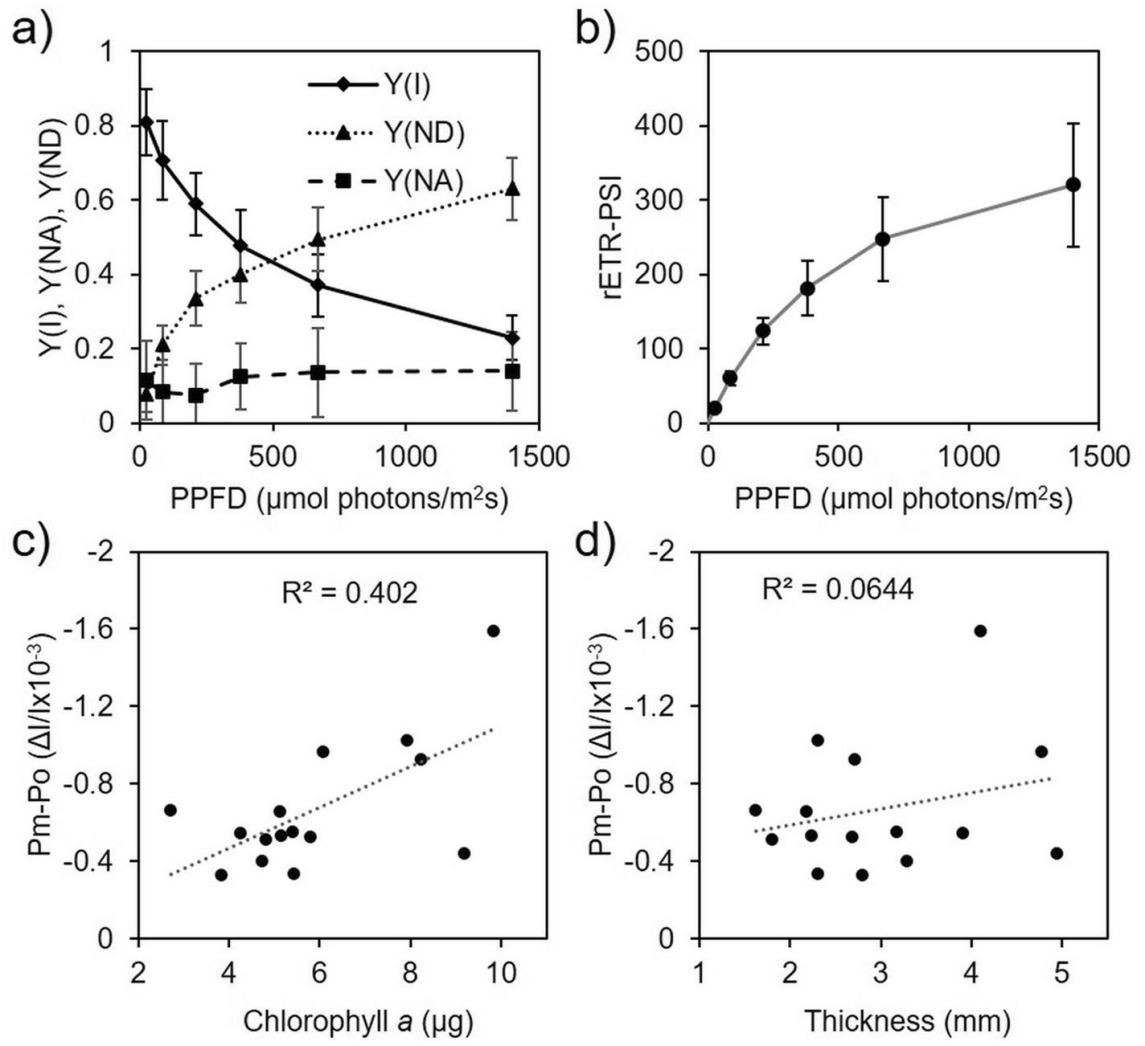
Light-dependent P700 oxidation measurement and PSI activity in *S. pistillata* coral fragments. (**a**) PSI quantum yield (Y(I), acceptor and donor side limitations (Y(NA) and Y(ND), respectively) as a function of PPFD). (**b**) Relative electron transport rate through PSI (rETR-PSI) calculated as the product of Y(I) and PPFD. (**c**,**d**) Maximum P700 absorbance change in the presence of 20 µM DCMU at high light (Pm) in function of its chlorophyll *a* content (**c**) or in function of its skeleton thickness (**d**). A correlation factor (R^2^) obtained from a linear trendline is shown for (**c**) and (**d**). n = 13 for (**a**) and (**b**).

We then tested the potential of coral fragments to monitor light-induced absorption changes of PSI primary electron donor (P700) in the far-red region of the visible spectrum (705 nm) (Fig. 1f). These absorption changes corresponding to P700 photo-oxidation are fully consistent with previous observations in plant leaves or algae^48^ and allowed us to calculate PSI quantum yield [Y(I)] and both PSI donor and acceptor side limitations [Y(ND) and Y(NA), respectively] at different light intensities (Fig. 5a). In addition, relative electron transport rates of PSI (rETR-PSI) were calculated from Y(I) values and a typical photosynthesis-light curve was obtained (Fig. 5b). We also observed a positive correlation (R^2^ = 0.40, *p* = 0.01) between absorbance changes upon a saturating pulse of light in presence of DCMU (Pm values) and chlorophyll *a* content per coral fragment (Fig. 5c). In contrast, thickness of coral fragments did not correlate (R^2^ = 0.06, *p* = 0.36) to the maximum amplitude of P700 absorbance change (Pm) (Fig. 5d). This indicates that the coral skeleton did not interfere significantly with our measurements.

**Figure 6:**
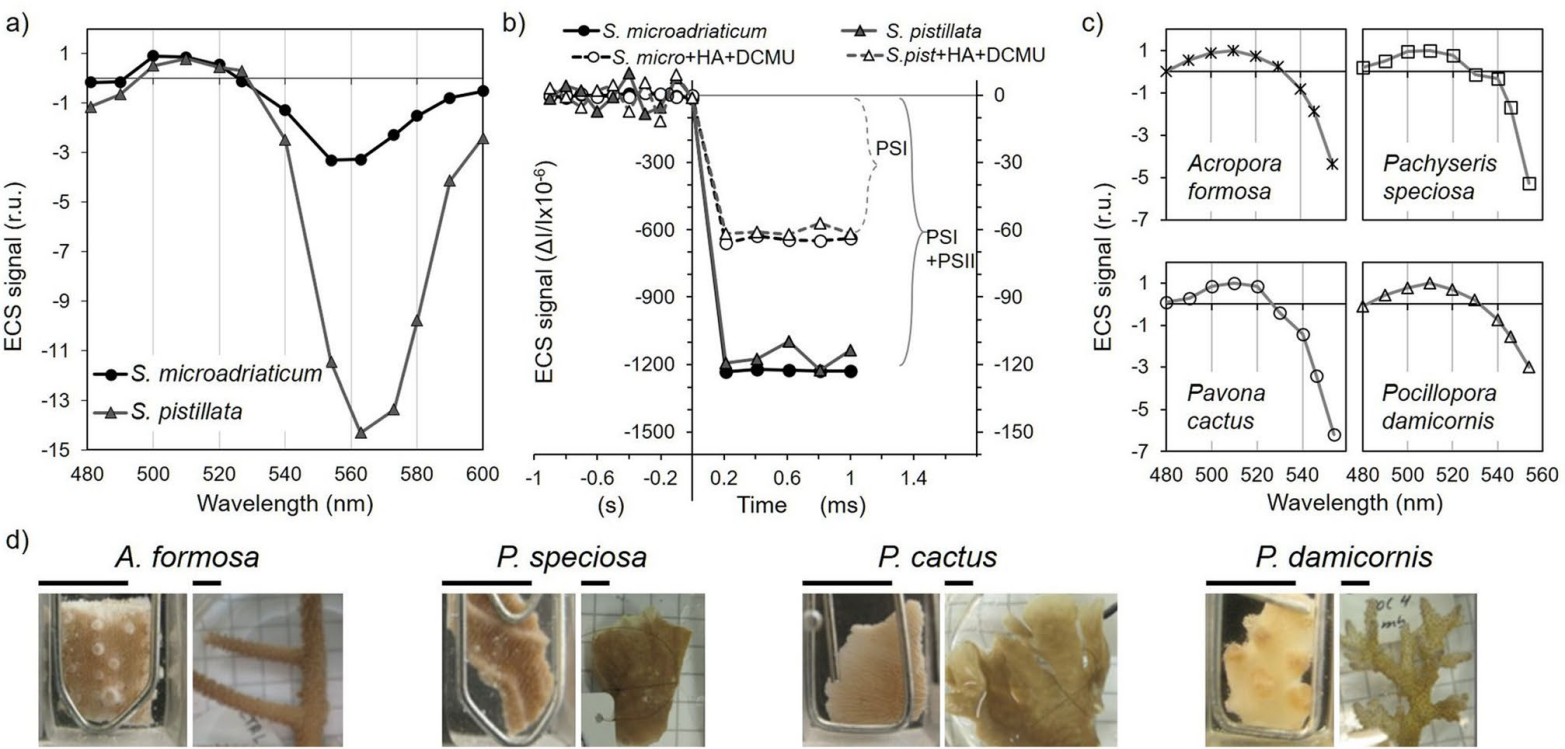
Electrochromic shift (ECS) signal from different Symbiodiniaceae sources. (**a**) ECS signal is compared between isolated and *in hospite S. microadriaticum* cells normalized to ECS at 510 nm (r.u. = relative units normalized to ECS signal at 510 nm). (**b**) PSI and PSII contribution to ECS signal at 554 nm was assessed by addition of PSII inhibitors hydroxylamine (HA, 1 mM) and DCMU (20 µM). Note the different scale magnitude for ECS signal between *S. microadriaticum* (left axis) and *S. pistillata* (right axis) acquisition. Time scale shows different units for negative and positive values. (**c**) ECS signal obtained from four different scleractinian corals species freshly sampled in the field (r.u. = relative units normalized to ECS signal at 510 nm). (**d**) True colour pictures of the coral fragments used to obtain ECS spectrum and a representative picture of the corals collected. The black bar stands for 5 mm. n = 3 for (**a**) and (**b**).

We next quantified the fraction of active PSI and PSII centers in coral fragments by monitoring the electrochromic shift signal (ECS), a technique previously used in various algal species, including Symbiodiniaceae^37,49^. The overall shape of the ECS spectrum of *S. pistillata* was similar to the one of its isolated symbiotic algae (*S. microadriaticum*), with a maximum and a minimum observed at 510 nm and around 560 nm, respectively (Fig. 6a). The ratio between maximum (510 nm) and minimum (560 nm) values was smaller in coral fragments than in isolated algae. We then assessed the amplitude of the fast ECS (< 1 ms) at 554 nm in response to a saturating single turnover flash (Fig. 6b). Under these conditions, the amplitude of the ECS is proportional to the amount of active PSI + PSII (reviewed in^49^). Addition of Hydroxylamine (HA) and DCMU, two PSII inhibitors, halved the amplitude of the ECS both in coral fragments of *S. pistillata* and isolated *S. microadriaticum* (Fig. 6b), indicating that PSI to PSII ratio is about 1 in both cases.

The four different coral species collected from Palau harbored the same *Symbiodiniaceae* genus (*Durusdinium* sp), which is a dominant algal genotype of inshore corals in this area^55^. For them, a similar ECS spectrum was obtained (Fig. 6c, d). It was not possible to test PSI:PSII stoichiometry in these coral species because a saturating flash laser was not available at that time of analysis.

**Figure 7:**
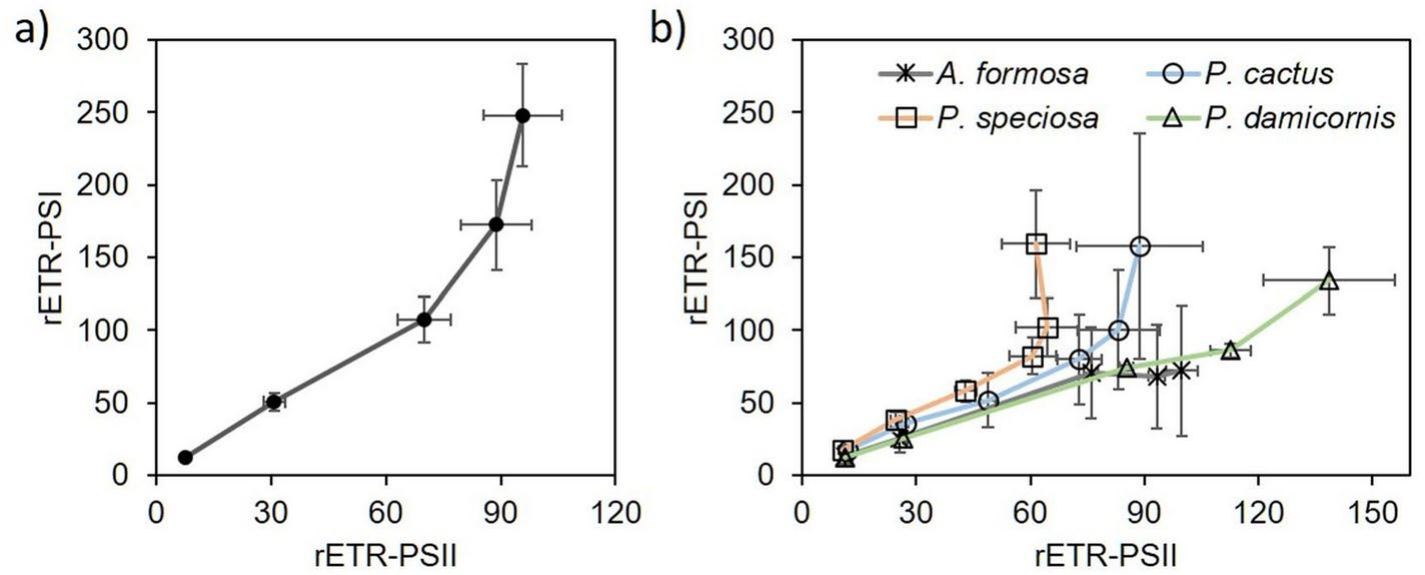
Relationship between rETR-PSI and rETR-PSII in *S. pistillata* (**a**) and in four reef-building corals species freshly collected in the field (**b**). Each light step was 3 min. n = 6 for (**a**), n = 5 for (**b**).

Spectroscopic-based photosynthesis activity measurements allowed us to compare different parameters obtained from small coral fragments. In *S. pistillata*, rETR-PSII linearly correlated (R^2^ = 0.999, *p* = 0.0005) to rETR-PSI up to the minimum saturating light intensity (around 200 µmol photons m^−2^ s^−1^), after which PSI activity continued increasing compared to PSII (Fig. 7a). Corals from the field revealed different responses when PSI and PSII activities were compared (Fig. 7b). A linear response was obtained from the two branching corals *A. formosa* (R^2^ = 0.95, *p* = 0.004) and *P. damicornis* (R^2^ = 0.96, *p* = 0.003) along with the different light intensities tested, but the laminar corals *P. cactus* and *P. speciosa* exhibited a disrupted relationship when light intensity was higher than 200 µmol photons m^−2^ s^−1^.

## Discussion

Coral survival to fragmentation is a well-known and broadly documented phenomenon (e.g.^56^). In the case of *S. pistillata*, a robust scleractinian coral species, small nubbins survive after fragmentation as well^54^ and have been used in skeleton calcification observations^57^. In this work, we demonstrate that thin coral fragments (around 25 mm^2^) can be used to study various bioenergetics traits which so far have not been assessed in reef-building coral species.

The chlorophyll *a* fluorescence-based photosynthetic parameter Fv/Fm, which accounts for the maximum quantum yield of PSII, has been used to track damages on the photosynthetic capacity and health in photosynthetic organisms, including corals (e.g.^58^). On the other hand, OJIP chlorophyll fluorescence rise has been used to reveal alterations of the PSII reaction center in Symbiodiniaceae cultures^59^ and changes in the redox state of PSII acceptor side in corals^60^. The fact that we did not observe any significant modification neither of the Fv/Fm value nor in OJIP chlorophyll *a* fluorescence kinetics up to 48 h after fragmentation in small coral fragments (Fig. 2), indicates the absence of structural or functional disturbance in the photosynthetic electron transfer chain. Moreover, our coral fragments exhibited a photoprotection capacity (NPQ; Fig. 3d) comparable to values registered from branches of *S. pistillata* collected from shallow waters in the Red Sea^61^ and from colonies maintained in aquarium conditions for several years^31^. Respiration measured in our coral fragments (Rd = 13.9 ± 2.1 nmol O_2_ cm^−2^ min^−1^; Fig. 3a) agreed with previous studies on 2 cm long nubbins or 10 to 15 cm long branches from *S. pistillata* collected in the Red Sea (11 nmol ± 4 O_2_ cm^−2^ min^−1^ in average)^12,62^. Maximum gross photosynthetic rate (Pgmax = 33 ± 4.7 nmol O_2_ cm^−2^ min^−1^; Fig. 3a) was also similar to values measured in coral branches of this species either maintained in aquarium conditions or freshly collected from the sea (20–35 nmol O_2_ cm^−2^ min^−1^)^31,62^. Rd and Pgmax values can be used to roughly calculate an extrapolated daily photosynthesis to respiration ratio (Pgmax/Rd*2)^63^. In our thin coral fragments of *S. pistillata* this ratio is 1.23 ± 0.16, a value in good agreement with *S. pistillata* branches (10 to 15 cm long) collected in summer from the Gulf of Aqaba (1.4)^56^, or from nubbins (2 to 3 cm long) prepared from *S. pistillata* coral colonies grown in aquarium conditions (1.1 to 1.5)^63^. Similarities between our results and data from the literature show that values measured on small thin fragments give reliable information on energetic relationships as those measured from bigger pieces of corals.

Alternative electron transport pathways in mitochondria and chloroplasts are described as regulators of the redox poise in the main electron transfer chains, and more generally are known for their influence on the energetic state of the cell^64^. By measuring oxygen consumption rates in *S. pistillata* in presence of inhibitors (Fig. 4), we evidenced, for the first time, the presence of a cyanide-insensitive but SHAM-sensitive mitochondrial alternative oxidase (AOX) activity in coral. In several algal species, AOX contributes to dissipate excess of redox equivalents produced in the chloroplast during photosynthetic activity (*i.e.* NAD(P)H) (e.g.^66^). The capacity of the AOX pathway measured in *S. pistillata* was 24% of Rd and is in the range as values previously reported for different species of *Symbiodiniaceae* grown in culture^20,65^. The observed lack of effect of the AOX inhibitor (i.e. SHAM) addition to fully respiring coral fragments demonstrates a high capacity of mitochondrial respiration through Complex IV. However, further studies are needed to determine the extent of both the cnidarian and photosymbiont’s AOX contribution to whole holobiont respiration and to elucidate the role of AOX under control and stress conditions.

Besides, in several species of Symbiodiniaceae in culture, a high light-dependent oxygen uptake was found to occur at the acceptor side of PSI through a Mehler-type reaction^37,38^. As illustrated in Fig. 3, the linear relationship between rETR-PSII and oxygen evolution observed in *S. pistillata* suggests that the photosynthetic electron transfer rate in high light does not depend on an alternative electron pathway to oxygen reduction as it does in free living cultures (e.g.^37,67^). This result may be explained by a lower CO_2_ limitation for symbionts *in hospite* compared to those isolated in culture^26,37^, but does not exclude that a different strategy operates under high light intensities. In this respect, PSI-dependent CEF is another alternative photosynthetic pathway that has been suggested to play an important role in the energetic balance in Symbiodiniaceae^23,36^. The most recommended way to assess CEF in vivo is to compare PSI and PSII activities^68^ (Fig. 7). Despite the fact that calculation of absolute electron transport was not assessed^69^ in the present study, our results indicate the occurrence of CEF in high light in *S. pistillata* and in two laminar corals from the field (*P. cactus* and *P. speciosa*). In contrast, we found that light influenced at the same proportion the relative electron transport rate of both photosystems in *A. formosa and P. damicornis*, two branching coral species, indicating that CEF is neglectable in high light (Fig. 7). It is worth to notice that these last four corals species harbour the same *Durusdinium* genus. A previous study, based on chlorophyll *a* fluorescence Serial Irradiation Pulses (SIP) method, indicated the absence of CEF in isolates of *Durusdinium* genus (referred as Clade D)^36^. Altogether, this highlights that involvement of photosynthetic alternative electron flows may strongly differ between corals harboring Symbiodiniaceae from the same genus.

Contrastingly to PSII, the role of PSI on photoacclimation has just started to be revealed in Symbiodiniaceae dinoflagellates^70^. PSI has been the target of very few studies on coral photosynthesis due to technical constraints. An indirect determination made on freshly extracted algae from *S. pistillata* showed a higher PSI content in coral colonies in symbiosis with *Symbiodinium* sp. (referred as Clade A) than *Cladocopium* sp. (referred as Clade C)^61^, suggesting that PSI content can be determinant for coral photosynthesis adaptation to changing light regimes. Other studies, aiming to assess in vivo PSI activity in nubbins from other coral species were performed by following changes in light reflectance of 820 or 830 nm, and correcting the signal at 870 nm with a pulse-amplitude-modulated Dual-PAM instrument (Walz, Germany)^39,40^. With this method a correction at 870 nm is commonly implemented in PSI activity determination to deconvolute PC contribution in the far-red region^48^. However, due to the absence of PC in peridinin chloroplasts^44^ we consider that this correction is not necessary in photosynthetic corals, and light-induced absorption change at 705 nm can be used (Fig. 1f), as it commonly yields a high negative value in plants^46^ and green algae^71^. In the case of Symbiodiniaceae isolates, absorbance change at 705 nm has been used with no major differences in kinetics of P700 photo-oxidation when a correction at 740 nm was applied (personal observation^37,38^). Assuming that the molar attenuation coefficient of P700 at 705 nm of Symbiodiniaceae is in the range of values determined for P700 in green photosynthetic organisms (i.e. 64 mM^−1^ cm^−1^ in spinach^72^ and 105 mM^−1^ cm^−1^ in *Chlamydomonas reinhardtii*^47^), we estimated the number of P700 per chlorophyll *a* to be between 1/720 to 1/1180 in *S. pistillata*. This range of values is in good agreement with values reported for several Symbiodiniaceae isolates^35,37^. Despite missing a molar absorption coefficient of P700 for peridinin plastids, our results indicate that spectroscopy-based measurement in thin coral fragments is a reliable and accurate strategy to ascertain PSI activity of coral endosymbionts (e.g. Fig. 5).

As thin coral fragments enabled spectroscopy-based measurements, ECS spectrum could be acquired. The presence of an ECS signal allows a direct measure of the electric field across algal thylakoid membranes^49^. The ECS spectrum measured in *S. pistillata* showed a small ratio between maximum (510 nm) and minimum (560 nm) ECS values in comparison to Symbiodiniaceae in culture (Fig. 6a^37^). On the other hand, field coral specimens showed different ECS signal amplitudes at 554 nm compared to 510 nm, despite harbouring the same Symbiodiniaceae genera *Durusdinium* (Fig. 6c). In general, this difference can be attributed to pigment composition, distribution and orientation along the thylakoid membranes^49^, but we cannot exclude that other effects such as differences in coral tissue or skeleton optical properties contribute to this difference. ECS-based measurements of PSI to PSII active center ratio in coral fragments gave comparable values to those previously reported in *Symbiodiniaceae* isolates (Fig. 6b^37,38^).

In conclusion, in vivo monitoring of photosynthesis and respiration, as well as PSI and PSII activities, of *S. pistillata* coral fragments resulted in robust and reliable information. We thus consider that coral fragments are good spectroscopic objects to obtain reliable information about coral photosynthesis. This approach does not consume large amounts of coral colonies or nubbins allowing to limit the loss of living samples when destructive measures are taken. This work paves the way towards a better understanding of the role of bioenergetics processes in coral symbiosis establishment and persistence.
